# Tendentious Paper—Titles and Wrong Conclusions Lead to Fear in the Population and Medical Overconsumption. Comment on Luchian et al. Subclinical Myocardial Dysfunction in Patients with Persistent Dyspnea One Year after COVID-19. *Diagnostics* 2022, *12*, 57

**DOI:** 10.3390/diagnostics12081825

**Published:** 2022-07-29

**Authors:** Patrik Vankrunkelsven

**Affiliations:** Cochrane Belgium, Belgian Centre for Evidence-Based Medicine (CEBAM), Department of Public Health and Primary Care, Katholieke Universiteit Leuven, 3000 Leuven, Belgium; patrik.vankrunkelsven@kuleuven.be

This week I read a press release from the University Hospital of Brussels with the title “30% of hospitalised COVID-19 patients have a heart defect one year after acute illness”. It is based on an article by ML Luchian [1]. The title of the article also claims “Subclinical Myocardial Dysfunction”. This study compares 23 patients with self-reported dyspnea and 43 without 1 year after hospital admission for COVID-19. The study reports the following echocardiographic differences: Compared to asymptomatic patients, patients with dyspnea presented lower LV constructive work (GCW): 2183.7 ± 487.9 vs. 2483.1 ± 422.4, *p* = 0.024; and a lower global work index (GWI): 1960.0 ± 396.2 vs. 2221.1 ± 407.9, *p* = 0.030. These differences may be significant, but the question is whether they indicate myocardial dysfunction, as stated in the title.

However, the GCW and GWI values of the two groups, both with and without dyspnea, do not deviate from the normal reference values, which are based on the NORRE study [2] and that were underpinned by the authors. These normal reference values for the myocardial work parameters are GCW (mmHg%) 2232 ± 331 with limits of normality of 1582–2881 and GWI (mmHg%) 1896 ± 308 with limits of normality of 1292–2505 [2]. For the normal GLS, the authors refer to a “recommendation of −18%” based on Farsalinos [3]. However, this is not a recommendation, and the value has been carelessly quoted, as Farsalinos suggests −18.0% to −21.5% as a reference.

If we compare the values of the two groups of patients with these reference values, we see that the values are all within the limits of the normal population. This is expected, as D’Andrea et al. demonstrated that global longitudinal strain (GLS) was only pathological in patients with COVID myocarditis and that, in any case, it improved after the administration of the correct therapy [4].

Thus, the only thing the article shows is that there is variation between patients with and without dyspnea one year after COVID-19, but there is no indication that there are any cardiac abnormalities. The title and conclusions of the article are therefore wrong. The media reverberations also lead to fear in the population and medical overconsumption. In my view, the article should be retracted or republished with the title “Patients with Persistent Dyspnea One Year after COVID-19 had a normal Myocardial Function”.
